# Supplementary material for: Multi‐dataset identification of innovative feature genes and molecular mechanisms in keratoconus
Source: J Cell Mol Med. 2024 Sep 19;28(18):e70079. doi: 10.1111/jcmm.70079 (PMC11412914; doi:10.1111/jcmm.70079)
Supplement: Supplementary file 2 — Material S2. [file JCMM-28-e70079-s002.zip › 7.GSEA analysis/5.RNF217-AS1/KEGG_PRIMARY_BILE_ACID_BIOSYNTHESIS.html]

Details for gene set KEGG\_PRIMARY\_BILE\_ACID\_BIOSYNTHESIS[GSEA]

|  || Dataset | RNF217-AS1.group.cls#h\_versus\_l |
| Phenotype | group.cls#h\_versus\_l |
| Upregulated in class | l |
| GeneSet | KEGG\_PRIMARY\_BILE\_ACID\_BIOSYNTHESIS |
| Enrichment Score (ES) | -0.5478087 |
| Normalized Enrichment Score (NES) | -1.2526121 |
| Nominal p-value | 0.19583334 |
| FDR q-value | 0.43109146 |
| FWER p-Value | 0.969 |
Table: GSEA Results Summary

  

Fig 1: Enrichment plot: KEGG\_PRIMARY\_BILE\_ACID\_BIOSYNTHESIS      
 Profile of the Running ES Score & Positions of GeneSet Members on the Rank Ordered List

  

| SYMBOL | TITLE | RANK IN GENE LIST | RANK METRIC SCORE | RUNNING ES | CORE ENRICHMENT || 1 | BAAT | na | 1973 | 0.352 | 0.0520 | No |
| 2 | CYP7A1 | na | 2728 | 0.304 | 0.1244 | No |
| 3 | CYP46A1 | na | 8359 | 0.120 | -0.0009 | No |
| 4 | SCP2 | na | 16565 | 0.003 | -0.2363 | No |
| 5 | HSD17B4 | na | 17409 | -0.006 | -0.2589 | No |
| 6 | HSD3B7 | na | 18270 | -0.013 | -0.2796 | No |
| 7 | SLC27A5 | na | 18784 | -0.018 | -0.2887 | No |
| 8 | AKR1C4 | na | 22318 | -0.054 | -0.3738 | No |
| 9 | AKR1D1 | na | 28360 | -0.158 | -0.4991 | Yes |
| 10 | CYP7B1 | na | 28648 | -0.166 | -0.4559 | Yes |
| 11 | CYP8B1 | na | 30652 | -0.228 | -0.4430 | Yes |
| 12 | ACOX2 | na | 30695 | -0.230 | -0.3732 | Yes |
| 13 | AMACR | na | 31156 | -0.248 | -0.3098 | Yes |
| 14 | CYP27A1 | na | 32740 | -0.341 | -0.2501 | Yes |
| 15 | CH25H | na | 33735 | -0.438 | -0.1432 | Yes |
| 16 | CYP39A1 | na | 34293 | -0.556 | 0.0125 | Yes |
Table: GSEA details [plain text format]

  

Fig 2: KEGG\_PRIMARY\_BILE\_ACID\_BIOSYNTHESIS      
 Blue-Pink O' Gram in the Space of the Analyzed GeneSet

  

Fig 3: KEGG\_PRIMARY\_BILE\_ACID\_BIOSYNTHESIS: Random ES distribution      
 Gene set null distribution of ES for **KEGG\_PRIMARY\_BILE\_ACID\_BIOSYNTHESIS**
